# Revisiting sequential attributable fractions

**DOI:** 10.1186/s13690-020-00442-x

**Published:** 2020-07-21

**Authors:** John Ferguson, Maurice O’Connell, Martin O’Donnell

**Affiliations:** HRB Clinical Research Facility, NUI Galway, Galway, Ireland

**Keywords:** Attributable fraction, Causal DAG, Do-operator, Bayesian network, Causal inference

## Abstract

**Background:**

In 1995, Eide and Gefeller introduced the concepts of sequential and average attributable fractions as methods to partition the risk of disease among differing exposures. In particular, sequential attributable fractions are interpreted in terms of an incremental reduction in disease prevalence associated with removing a particular risk factor from the population, having removed other risk factors. Clearly, both concepts are causal entities, but are not usually estimated within a causal inference framework.

**Methods:**

We propose causal definitions of sequential and average attributable fractions using the potential outcomes framework. To estimate these quantities in practice, we model exposure-exposure and exposure-disease interrelationships using a causal Bayesian network, assuming no unmeasured latent confounders. This allows us to model not only the *direct* impact of removing a risk factor on disease, but also the *indirect* impact through the effect on the prevalence of causally downstream risk factors that are typically ignored when calculating sequential and average attributable fractions. The procedure for calculating sequential attributable fractions involves repeated applications of Pearl’s do-operator over a fitted Bayesian network, and simulation from the resulting joint probability distributions.

**Results:**

The methods are applied to the INTERSTROKE study, which was designed to quantify disease burden attributable to the major risk factors for stroke. The resulting sequential and average attributable fractions are compared with results from a prior estimation approach which uses a single logistic model and which does not properly account for differing causal pathways.

**Conclusions:**

In contrast to estimation using a single regression model, the proposed approaches allow consistent estimation of sequential, joint and average attributable fractions under general causal structures.

## Background

As has been noted elsewhere, confusion abounds regarding the definition and interpretation of population attributable fractions (PAF) in epidemiology [1]. For instance, in their seminal paper where Eide and Gefeller introduce average and sequential attributable fractions [2], they define the population attributable fraction as ‘the proportion by which a disease prevalence (or incidence) is reduced if the whole population is hypothesized to attain the same risk of disease as the individuals within the lowest exposure category.’ The problem with such a definition is it is non-causal. That is, if individuals in the lowest exposure category do have a lower disease risk, it might not be because of any health benefit attributable to the exposure, but because of spurious correlations or even reverse causation. Taking this kind of logic to the extreme, one could make quite non-sensible conclusions regarding say the cot-death risk attributable to Swiss cheese consumption, or the risk of heart disease attributable to doctor visits. Of course, Eide and Gefeller clearly understand this, and later in the paper mention that ‘if there exists a direct cause-effect relationship between the exposure and the disease, the attributable fraction may be interpreted as the proportion of the diseased that would have been prevented if the exposure was totally eliminated from it’ (note that the use of the word eliminate is convenient but slightly misleading as it refers to a hypothetical population where the risk factor of interest was *always absent* rather than eliminated at a point in time). They then define this second quantity as the ‘etiologic fraction’, introduced by Miettinen [3]. Incidentally, Robins and Greenland [4] discuss a subtly different metric, more directly interpretable as the proportion of disease caused by a risk factor which they also call an etiologic fraction. More recently, the epidemiological community seems to have settled on Miettinen’s definition ([5, 6]). This seems sensible to us as it does have a direct causal implication (that is, it will only be non-zero if the exposure has some causal effect on disease), and can be estimated in real data, provided we can adequately adjust for confounding [7].

While the standard definition of an attributable fraction only pertains to one risk factor, sequential and average attributable fractions [2] focus on measuring the cumulative contribution of a collection of risk factors to disease and indeed on partitioning this quantity into individual contributions for each risk factor. In particular, a sequential attributable fraction for a risk factor can be informally defined as the relative change in disease prevalence due to removing the risk factor from the population in a situation where a subset of the other risk factors under investigation are already eliminated; different sequential attributable fractions corresponding to differing risk factor elimination orders (note again that ‘removal’ technically refers to disease risk in hypothetical populations where all risk factors in a particular set were always absent). A crucial point to consider when estimating this quantity is that risk factors have effects on each other as well as effects on disease. As an example, a dietary intervention might effect cholesterol and blood pressure; that is there may be direct effects on disease as well as effects of disease mediated through other risk factors. This implies that the sequential attributable fraction for blood pressure, having first intervened on diet would be different from the population attributable fraction for blood pressure because:

1. Some disease cases, that would occur under a no-intervention scenario, may have been prevented by the prior intervention on diet. The subsequent intervention on blood pressure will have no impact on these individuals.
2. The intervention on diet has changed the distribution of blood pressure in the population. This will change the impact of a subsequent intervention on hypertension

Usually calculations of sequential attributable fractions (including the author’s own R-package, *averisk* as well as the calculations demonstrated in [8]) properly incorporate (1), but don’t allow for (2) and as a result can generate biased estimates of sequential disease burden, although some proposals have been suggested to deal with this issue from a non-causal perspective ([9]). As an alternative, here we describe a Monte-Carlo approach based on a causal Bayesian network describing the inter-relationships between all risk factors and disease. This approach promises consistent estimation of sequential attributable fraction and average attributable fractions under any known causal graph, with a proviso that statistical models are also correctly specified. Interestingly, while sequential attributable fractions can be substantially biased when causal structure is ignored, average attributable fractions might be affected to a lesser degree as positive and negative biases for various sequential fractions may partially cancel. Empirical evidence for this observation is shown in the Results section using, INTERSTROKE [10], an international case control dataset used to investigate the contributors to stroke on a global level.

## Methods

### Causal definitions of attributable fractions in a multi-risk factor setting

Let *Y*_*i*_∈{0,1} be the observed disease outcome for an individual, labeled *i*, selected from the population. Suppose there are K risk factors or exposures that might effect disease status; risk factors could be binary (eg. diabetes Y/N), categorical (current, previous or never for smoking status) or continuous (blood pressure). The observed values of these *K* risk factors for person *i* are denoted $A_{i}^{1},A_{i}^{2},...,A_{i}^{K}$. To define population attributable fractions in a causal framework, some counterfactual notation [11] is necessary. In this regard, we define

1 $$ \begin{aligned} Y_{i}\left(a^{1},a^{2}...,a^{K}\right) \end{aligned}  $$

as the potential disease outcome for person *i* in a world where we had somehow intervened on all *K* risk factors to set $A_{i}^{1}=a^{1},...,A_{i}^{K}=a^{K}$, with *a*^1^,...,*a*^*K*^ possible values for the *K* risk factors. Note that we will sometimes write (1) as *Y*_*i*_(**A**=(*a*^1^,*a*^2^...,*a*^*K*^)), a notation that will be more convenient when we intervene only on a subset of the risk factors. It is important that we also define potential outcomes for subsets of the risk factors. For shorthand convenience, we denote the observed vector $\{A_{i}^{k};k \in \mathbf {S}\}$ as *A*_*i*,*S*_. In this case, we can consider potential outcomes for the remaining risk factors, *S*^*c*^=1,...,*K*∖**S**, as $\phantom {\dot {i}\!}\mathbf {A_{i,S^{c}}}(\mathbf {A_{S}=a_{S}})$, if *A*_*i*,*S*_=*a*_*S*_.

The disease indicator for person *i* based on the intervention on *A*_*i*,*S*_ is now $\phantom {\dot {i}\!}Y_{i}(\mathbf {A_{S}}=\mathbf {a_{S}},\mathbf {A_{S^{c}}}=\mathbf {A_{i,S^{c}}}(\mathbf {A_{S}=a_{S}}))$. Note that the contributions before and after the comma in the preceeding expression in some way denote the direct and indirect effects of *A*_*S*_. However, if we fix individual *i*, the expression is just a function of *a*_*S*_, and so we will write this informally as *Y*_*i*_(*a*_*S*_), in a slight abuse of notation (that is technically, the new function *Y*_*i*_ is no longer the same function as in (1). Suppose that *a*_*S*_=0_*S*_ represents the reference level of the risk factor. Imagine intervening on risk factor *j*∈*S*^*c*^ having already made the intervention: $\mathbf {A_{i}^{S}}=\mathbf {0_{S}}$; it follows immediately that the potential outcome for the new joint intervention changes to: *Y*_*i*_(0_*S*∪*j*_). Treating *i* as a randomly selected individual from the population, a sequential attributable fraction for risk factor *j*, after a population intervention setting *A*_*S*_=0_*S*_ is then defined as:

2 $$ SAF_{j\vert\mathbf{S}}=\frac{P(Y(\mathbf{0_{S}})=1)-P(Y(\mathbf{0_{S\cup{j}}})=1)}{P(Y=1)}  $$

Now suppose that **S**=**ϕ**, the empty set. We define *Y*_*i*_(0_*ϕ*_)=*Y*_*i*_, the observed disease indicator under no intervention. It follows that:

3 $$\begin{array}{*{20}l} SAF_{j\vert\mathbf{\phi}} & =\frac{P(Y(\mathbf{0_{\phi}})=1)-P(Y(\mathbf{0_{\{j\}}})=1)}{P(Y=1)}\\  &=\frac{P(Y=1)-P(Y(\mathbf{0_{\{j\}}})=1)}{P(Y=1)}\\  &=PAF_{j} \end{array} $$

so that the above framework covers the regular attributable fraction. In addition, joint attributable fractions, which compare current disease prevalence with the hypothetical disease prevalence if a set of risk factors were removed (for instance an attributable fraction referring to a hypothetical population where nobody smoked or drank alcohol) can be defined as:

4 $$\begin{array}{*{20}l} PAF_{\mathbf{S}} & =\frac{P(Y=1)-P(Y(\mathbf{0_{S}})=1)}{P(Y=1)}, \\   \end{array} $$

which can be seen mathematically to equal the sum of the sequential attributable fractions, (2), corresponding to elimination of individual risk factors in *S*, no matter in which order the risk factors are eliminated. Note that this invariance property (that the sum of sequential attributable fractions is invariant to the order of elimination) is not an assumption, but actually a consequence of the causal definitions of sequential and joint attributable fractions given in this manuscript which compare disease prevalence in the real world and in a counterfactual world where a set of risk factors never existed (for instance, a world where tobacco and alcohol didn’t exist). As an aside, note that one could also define sequential attributable fractions using Pearl’s Do-algebra [12], with similar notation for risk factors and outcome, but identifying counterfactual probabilities *P*(*Y*(0_*S*_)=1) as *P*(*Y*=1|*do*{**A**_**S**_=0_*S*_}), with the do-notation indicating a probability distribution associated with the intervention **A**_**S**_=0_*S*_.

### Causal bayesian networks and framework for estimation

Suppose that $\mathcal {G}$ is a causal Bayesian network, informally a directed acyclic graph (DAG) where arrows representing causal dependencies between confounders, risk factors/exposure and disease, together with a sensible probability distribution on the graph that respects these causal dependencies. To consistently estimate causal effects that risk factors may have on each other and on disease, we need to make a strong no unmeasured confounding assumption: that is common causes of nodes in the graph, which may be causes of two risk factors or a cause of risk factor and disease, are also included as nodes in the graph within $\mathcal {G}$. Causal Bayesian networks have a local Markov property that the conditional probability distribution of any node *X*_*j*_, given values for the other variables in the network, only depends on the values $\boldsymbol {x_{pa_{j}}}$ of the parent nodes (here we assume that *pa*_*j*_⊂{1,..,*j*−1} - such a labeling of nodes can always be chosen for a DAG). That is:

5 $$\begin{array}{*{20}l} p_{X_{j} \vert \boldsymbol{X_{-j}}}(x_{j}\vert \boldsymbol{x_{-j}})=p_{X_{j} \vert \boldsymbol{X_{pa_{j}}}}(x_{j}|\boldsymbol{x_{pa_{j}}}) \end{array} $$

, implying that:

6 $$\begin{array}{*{20}l} & p_{X_{1},...,X_{N}}(x_{1},...,x_{N}) \\ & = \prod_{i \leq N}{p_{X_{i} \vert X_{1},..,.X_{i-1}}(x_{i}|x_{1},...,x_{i-1})} \\ & = \prod_{i \leq N}{p_{X_{i} \vert \boldsymbol{X_{pa_{i}}}}(x_{i}|\boldsymbol{x_{pa_{i}}})}.  \end{array} $$

A powerful feature of this framework is that the representation (6) extends easily to distributions derived from interventions. For example, intervening on risk factor *j* to eliminate it from the population is equivalent to removing the arrows into *j* in the corresponding causal graph. A key assumption here is that while this intervention does change the marginal distribution of *j* in the population, it doesn’t change any of the mechanisms by which risk factor *j* might effect other risk factors; or in other words that the conditional distributions $p_{X_{i} \vert \boldsymbol {X_{pa_{i}}}}(x_{i}|\boldsymbol {x_{pa_{i}}})$ remain unchanged for *i*≠*j*. Multiplying these conditionals leads to the joint distribution of {*X*_1_,...,*X*_*N*_} under this intervention (that sets *X*_*j*_=0):

7 $$ \begin{aligned} p_{X_{1},...,X_{N}|do\{X_{j}=0\}}(x_{1},...,x_{N}) = \prod_{i \leq N, i \neq j}p_{X_{i} \vert \boldsymbol{X_{pa_{i}}}}(x_{i}|\boldsymbol{x_{pa_{i}}})\mathbb{I}\{x_{j}=0\}. \end{aligned}  $$

The ‘do’ notation was originally introduced by Judea Pearl [12] to represent intervention distributions that are estimable from observational data. Our procedure here is to use (7) recursively to simulate sequential attributable fractions, using estimates of the conditional probability distributions: $\hat {p}_{X_{i} \vert \boldsymbol {X_{pa_{i}}}(x_{i}|\boldsymbol {x_{pa_{i}}})}$ fitted from real data. As a concrete example that should illustrate the main ideas, suppose that we have a causal graph and associated probability distribution (6), summarizing the causal relationships between smoking, high blood pressure (HBP) and disease. We refer to this dataset as *D*_*ϕ*_. We are interested in estimating the sequential attributable fractions: *SAF*_HBP|**ϕ**_, and *SAF*_Smoking|**{**HBP**}**_, based on a random sample of individuals *i*=1,...,*N*, with risk factors and disease generated according to (6). To estimate the first sequential attributable, we can simulate values {smoking_*i*_,HBP_*i*_,disease_*i*_} for each individual *i* from (7) where *j* represents ‘HBP’, that is from the intervention distribution corresponding to ‘do: HBP’=0. In this process, we only need to simulate descendants of the node that is fixed, since the marginal distribution of the ancestors of HBP are the same in (6) and in (7). This simulation produces a new (and random) dataset, *D*_*HBP*_, that consists of plausible values of {smoking_*i*_,HBP_*i*_,disease_*i*_} for each person under the intervention: ‘do: HBP=0’. Provided the number of individuals, *I* is large enough, one can estimate *SAF*_HBP|**ϕ**_ by the quantity: $\hat {SAF}_{\text {HBP}\vert \mathbf {\phi }} = \frac {\sum {Y_{i}}-\sum {D_{HBP}(Y_{i})}}{\sum {Y_{i}}}$, where *Y*_*i*_ denotes the disease status for individual *i* in the original dataset, and *D*_*HBP*_(*Y*_*i*_) the randomly assigned disease status for person *i* in *D*_*HBP*_. Note that this simulation based process takes into account both the direct effects of a blood pressure intervention as well as indirect effects through mediating risk factors. Next, we apply a second do-operator, corresponding to the intervention ‘do{smoking=0}’. This implicates simulating values for the descendents of smoking in a second Bayesian network, where both HBP and smoking are set to 0 with the non-descendants of smoking being fixed as in *D*_*HBP*_. The resulting dataset will be denoted *D*_{*Smoking*,*HBP*}_, with corresponding simulated values of disease: *D*_*Smoking*,*HBP*_(*Y*_*i*_). An estimate of the sequential attributable fraction for smoking, given an intervention that has eliminated hypertension is then: $\hat {SAF}_{\text {Smoking}\vert \mathbf {\{HBP\}}} = \frac {\sum {D_{HBP}(Y_{i})}-\sum {D_{\{Smoking,HBP\}}(Y_{i})}}{\sum {Y_{i}}}$. Sequential attributable fractions, that condition on the elimination of 2 or more risk factors are calculated similarly in a recursive fashion. For instance, the sequential attributable fraction corresponding to an intervention on *j*, having previously intervened on the set of risk factors **S** is given by:

8 $$\begin{array}{*{20}l} \hat{SAF}_{j \vert \mathbf{\{S\}}} = \frac{\sum{D_{S}(Y_{i})}-\sum{D_{S \cup j}(Y_{i})}}{\sum{Y_{i}}}  \end{array} $$

### Average attributable fractions

As described in [13] the average attributable fraction for a risk factor *j* represents the average sequential attributable fraction for *j* over all possible risk-factor elimination orders. For a large number of risk factors, *K* the number of elimination orders grows exponentially as *K*!, and calculating sequential attributable fractions for all possible orders quickly becomes infeasible. However, as demonstrated in that paper, one can approximate the average attributable fraction by randomly sampling elimination orders, calculating sequential attributable fractions for each risk factor according to each sampled elimination order, and finally averaging these over all sampled elimination orders. Here, we follow the same process of randomly sampling elimination orders, with the exception that at each step each sequential attributable fraction is subject to an additional Monte Carlo error (according to simulating realizations of the Bayesian network); fortunately, one can effectively eliminate this Monte Carlo error by simulating a sufficient number of elimination orders. In [13] we suggested average at least 1,000 randomly sampled elimination orders to calculate sequential and average attributable fractions with reasonable accuracy.

## Results

### INTERSTROKE project

We have previously used data from the INTERSTROKE project to illustrate methodologies for approximating average attributable fractions, [13]. Briefly, INTERSTROKE was a standardized international study of stroke cases and controls in 32 countries in Asia, Europe, Australia, the Middle East and Africa. In the original study, stroke cases were matched with controls according to age, gender and region. Here, we have restricted to the ischemic stroke patients and their matched controls. Interviews with hospital and community controls and stroke patients (or proxy respondents) post-stroke were used to retrospectively collect information on key causal and modifiable risk factors for stroke, as described in [10]. The risk factors examined were healthy eating score (in tertiles), physical inactivity (yes/no), smoking behaviour (current smoker or ex/no smoker), alcohol intake (no alcohol, moderate consumption, high consumption), an indicator for stress, ApoB/ApoA lipid ratio (in tertiles), pre-existing hypertension or high measured blood pressure (yes/no), waist hip ratio (in tertiles), cardiac risk factors such as atrial fibrillation or flutter (yes or no) and a diagnosis of diabetes mellitus or elevated HbA1c(yes/no). While the drawbacks of categorizing exposures into risk factors are well understood [7], we have repeated the 2016 analysis again with categorized risk factors firstly to enable comparability with our previous analysis and secondly since there are difficulties defining attributable fractions with continuous exposures, particularly when the relationship between exposure and disease risk is monotonic.

### A causal graph for INTERSTROKE

**Fig. 1:**
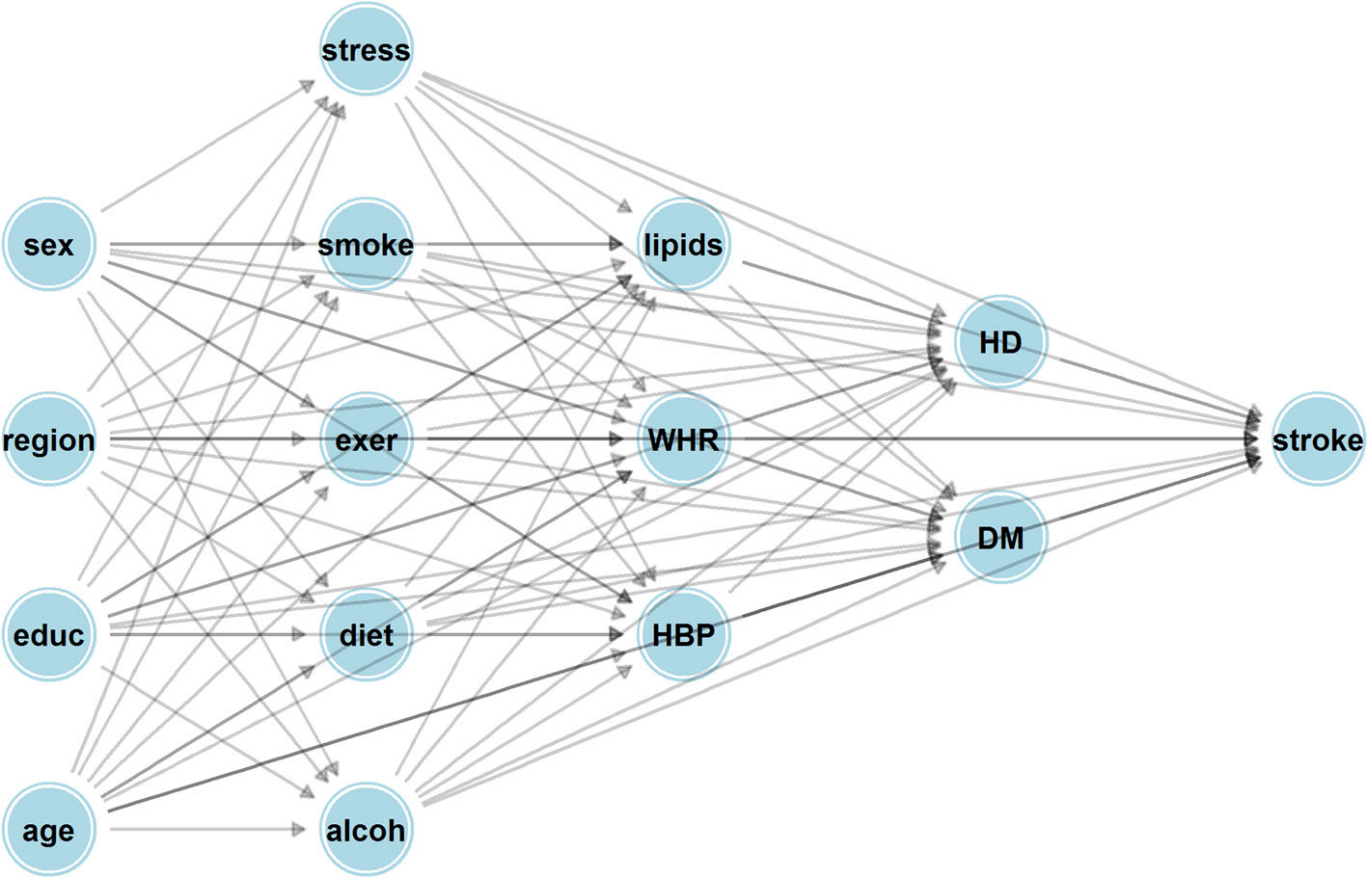
Hypothesized causal Bayesian network describing direct and indirect effects pertaining to causal risk factors and associated confounders for stroke. Abbreviations for variables in the causal graph are as follows. Sex: gender of participant; Region: Geographic area of participant either Western Europe, North America, Africa, South Asia, China, South America and South East Asia; Educ: years of education (None, less than 8, 9-12, more than 12); Stress: Summary variable for psychological stress (yes or no); Smoke: smoking status (current, ex-smoker, never smoker); Diet: AHEI diet score (in tertiles); Exer: physically active (yes or no); Alcoh: alcohol consumption (none; moderate; binge drinker); lipids: Apolipoproterion B/Apolipoprotein A1 ratio (in tertiles); WHR: waist hip ratio (in tertiles); HBP: clinically diagnosed high blood pressure (yes or no); HD (history of risk factors for heart disease - yes or no); DM (clinically diagnosed diabetes mellitus or measured Hba1c level at least 4.5 - yes or no)

**Fig. 2:**
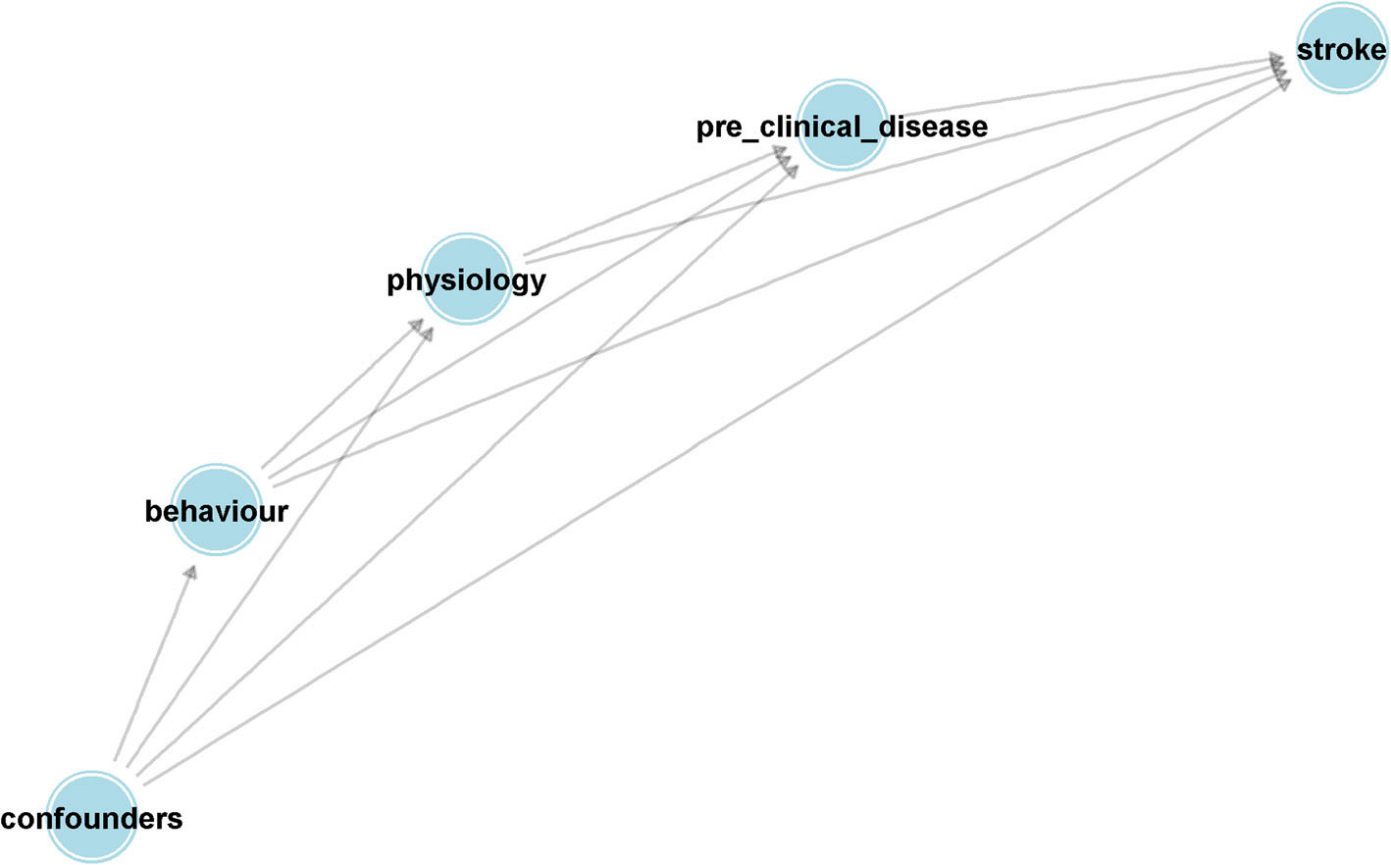
Simplification of network from Fig. 1, showing it’s layered structure. Confounders consists of the variables sex, region and education. Behaviour consists of the variables smoking status, diet, alcohol consumption, stress levels and physical activity. Physiology groups the variables lipids, waist hip ratio and high blood pressure. Pre-clinical disease consists of diabetes and risk factors for heart disease

One needs to assume a causal graph describing the causal relationships between confounders, risk factors and disease to implement the methods described earlier. To do this, it is helpful to divide the risk factors and exposures into categories depending on whether they are descriptive of an individual’s behaviour *S*_*B*_={Smoking, Alcohol intake, inactivity, diet and stress}, their physiology *S*_*P*_={High blood pressure, ApoB/ApoAratio, Waist hip ratio} and what might be regarded as pre-clinical disease *S*_*D*_={Cardiac risk factors, Pre-clinicaldiabetes}. We also consider a set of variables that might be confounders (joint causes of the risk factor and stroke) for all the listed risk factors. This set of confounders, *S*_*C*_ consist of the individuals and their parents’ education level (in 5 levels from no-education to holding a college Degree), age, gender and region. Here we make the simplifying assumption that disease develops in a stage-wise fashion, each stage being represented by one of the sets of variables just described with variables in earlier stages having causal effects on variables contained in later stages, but not vice-versa. The ordering of stages is indicated by the sequence, {*S*_*C*_,*S*_*B*_,*S*_*P*_,*S*_*D*_,*Y*}, and summarized by the causal graphs in Figs. 1 and 2.

### Estimation of probability models

The next step in the process is to estimate probability models corresponding to non-root nodes in Fig. 1. The process here is to simply fit logistic or proportional odds models depending on whether the target variable is binary or ordinal and adjusting for the variables that ‘point’ to the target node in question. For simplification, only adjustment for main-effects are made in the example here. More generally, more complicated models, possible incorporating general interaction structures could and should be used if necessary. To fit these models to case control data, one needs to perform weighted maximum-likelihood estimation to imitate estimation using a random sample from the population. We chose weights of 0.0035 (for each case) and 0.9965 (for each control), reflective of a yearly incidence of first ischemic stroke of 0.35%, or 3.5 strokes per 1,000 individuals. These weights were chosen according to average incidences across country, age group and gender within INTERSTROKE according to the global burden of disease [14]. In reality, the estimates are quite robust to the precise value of the case/control weight, as shown in the Additional file 1 of [13]. As a side note, we had first considered individually weighting each case to reflect incidence within a particular age/gender and region bracket, but the variability in the individual weights for each case transferred to increased variance in estimation in regression parameters, so the more crude correction was used instead.

### Estimation of sequential and average attributable fractions

We randomly sampled 10,000 elimination orders (or random permutations of the 10 risk factors) computing Monte-Carlo sequential attributable fractions for each random permutation. Similarly to the calculations in [13], a correction needs to be made to (8) when estimating sequential attributable fractions for case control structure; that is for a particular elimination order and Monte Carlo simulation, the corrected formula is:

9 $$\begin{array}{*{20}l} \hat{SAF}_{j \vert \mathbf{\{S\}}} = \frac{\sum{w_{i}D_{S}(Y_{i})}-\sum{w_{i}D_{S \cup j}(Y_{i})}}{\sum{w_{i}Y_{i}}}. \end{array} $$

, where the weights, *w*_*i*_ are described in the paragraph above. In Fig. 4, we investigate how the mean of these estimated sequential attributable fractions depends on the position in the elimination order for two causal graphs:

1. Bayesian network model with direct and indirect effects, represented by Fig. 1 and summarized by Fig. 2.

**Fig. 3:**
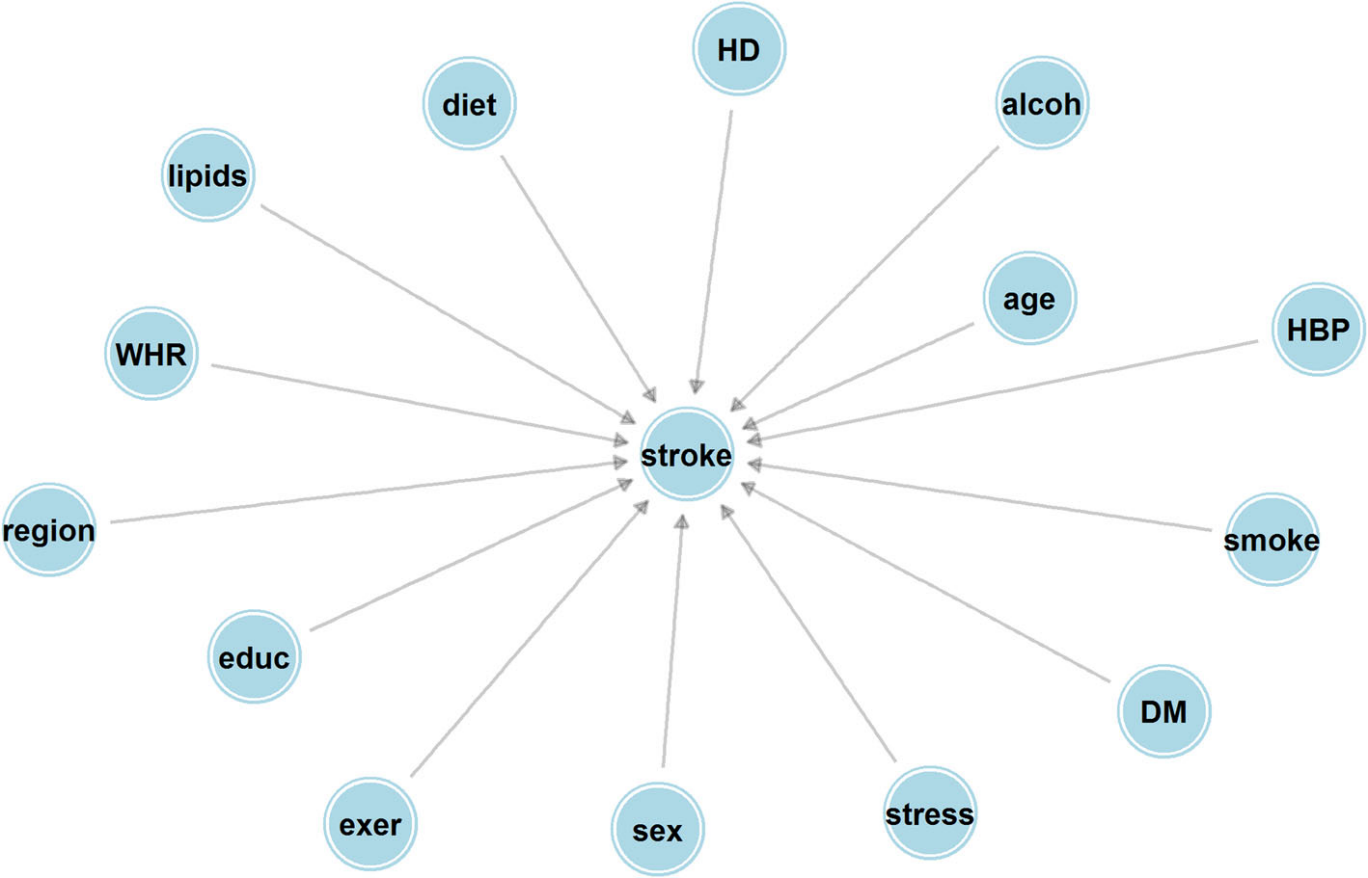
Bayesian network with only direct effects. Abbreviations for nodes are as listed in Fig. 1

**Fig. 4:**
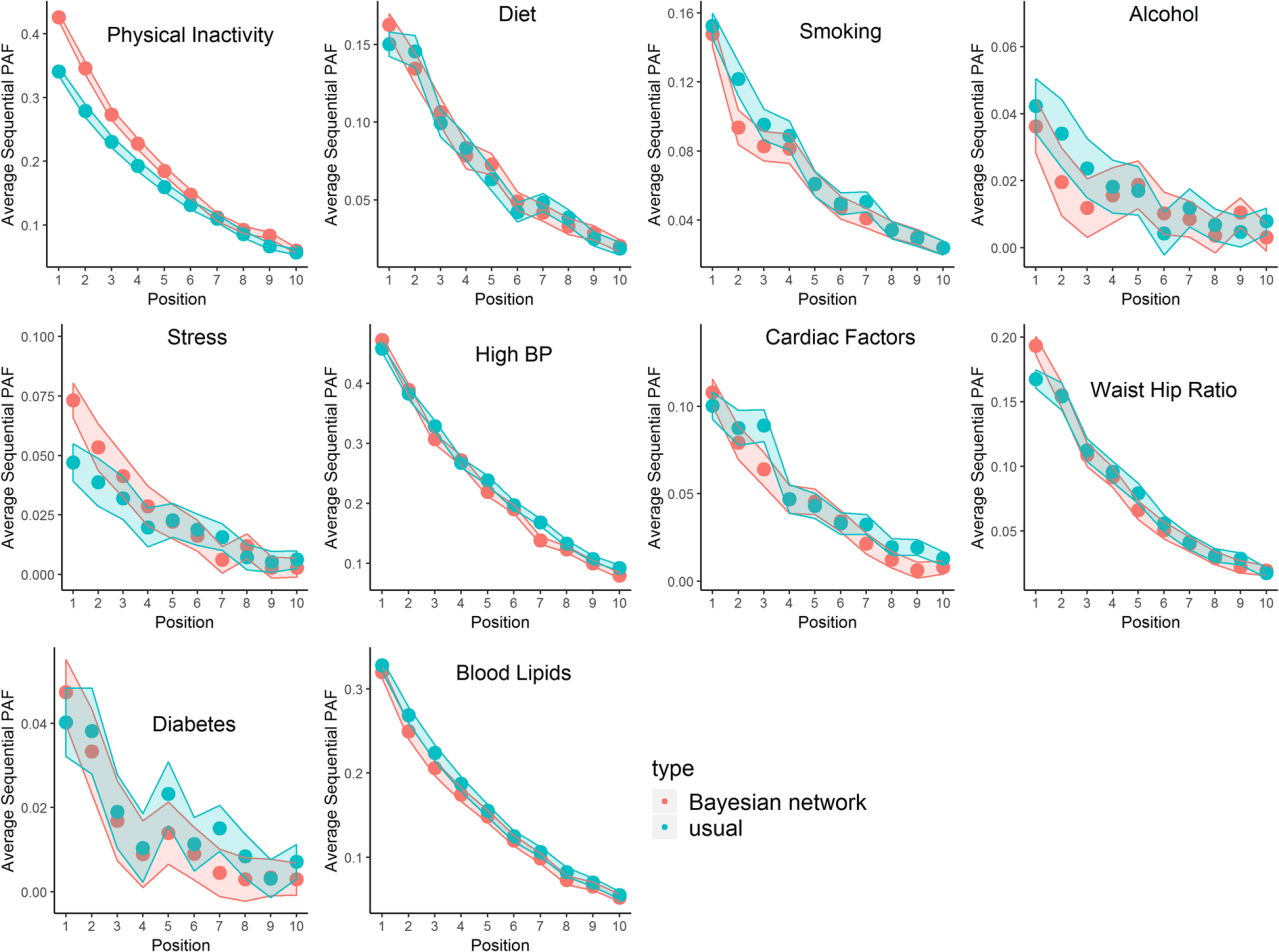
Estimated sequential attributable fractions, by position in elmination order. We can be 95% confident the true estimate (that would be calculated from the procedure when the number of simulations *m*→*∞*) lies in the Monte Carlo interval around the point estimate. The estimates shaded red correspond to the Bayesian network in Fig. 1, whereas the estimates shaded blue correspond to the Bayesian network in Fig. 3. Note that the Monte Carlo error at position *k* incorporates variation due to random selection of the set of risk factors/exposures that are intervened on in stages 1,... *k*−1, and also variation based on the recursive simulation of the disease response described in the main text
2. A Bayesian network model with direct effects only represented by Fig. 3.

In particular, the second graph represents a model where there are no indirect effects of a risk factor on stroke, which is effectively assumed in previous approaches for calculating sequential attributable fractions which used a single logistic model. The most prominent feature of Fig. 4 is the difference in sequential attributable fractions for physical inactivity when eliminated first (the sequential attributable fraction being higher under Fig. 1). This might be something that we would expect a-priori since physical activity should have beneficial effects on downstream risk factors such as blood pressure and waist hip ratio and these indirect effects may reduce stroke risk (Recall that Fig. 3 only considers direct effects, whereas Fig. 1 considers both direct and indirect effects). Perhaps less intuitively, the sequential attributable fraction for alcohol in positions 1, 2 and 3 are higher using the model that only considers direct effects. A naive interpretation might be that the indirect effects of eliminating alcohol result in increased stroke risk. Examining the fitted models, we see that the intermediate pathways involving alcohol are ambiguous. Binge drinking almost halves the odds of being in the top lipid (APOB/APOA) tertile (a 44% reduction to be precise) compared to a non-drinker, but increases the odds of hypertension by 65%, has no appreciable effect on waist hip ratio (the point estimate indicates an increase of 9.8% in the odds of the top tertile), reduces the odds of cardiac risk factors by 11.5% and has no appreciable effect on the effect of diabetes (a 7.5% increase in odds). In summary, these intermediate pathways seem to somewhat attenuate the direct effect of alcohol on stroke (the direct effect of binge drinking is to increase stroke risk by 82.8%, according to the fitted probability distribution for stroke), and slightly reduce the sequential attributable fraction for alcohol, at least when alcohol is one of the first risk factors to be eliminated (The estimated odds ratios corresponding to the inter-relationships between risk from Fig. 1 are given as Additional file 2). Here it should be emphasized that these effects are only as good as the causal graph and statistical models that were a-priori assumed, and these are surely at best rough approximations to the truth. In particular, it is possible that reverse causation might be at play; for instance, the negative correlation between alcohol consumption and cardiac factors might be explained by individuals changing their alcohol consumption post diagnosis of atrial fibrilation. It is the estimation approach, rather than the exact values of the estimates that we would like to emphasize here. Average attributable fractions for the two causal graphs are reported in Table 1. The total estimated PAF for eliminating all 10 risk factors is 88.6% for both causal structures, found by summing the average attributable fractions across all risk factors. Similarly to the case with sequential fractions, the average attributable fractions are higher for physical inactivity and stress when incorporating indirect pathways via the Bayesian network, and higher for alcohol intake when ignoring indirect pathways, although these differences are relatively smaller for average PAF than for sequential PAF. The Monte Carlo standard error is between 0.1% and 0.2%, indicating that 10,000 simulations is more than sufficient to provide a good aproximation to the true estimate. Note that these standard errors (and the error bands in Fig. 4) display Monte Carlo error; bootstrapping the entire procedure is necessary to estimate confidence intervals for average attributable fractions.

**Table 1:** Average attributable fractions and standard errors for 10 INTERSTROKE risk factors. BN (Bayesian network) corresponds to the causal structure shown in Fig. 1, whereas DE (Direct effects only) corresponds to the causal structure in Fig 3. The Monte Carlo SE is reported for the estimates in the top row of the table, but would be similar for the estimates corresponding to Fig. 3

	Inactivity	Diet	Smoking	Alcohol	Stress	High BP	Lipids	WHR	Cardiac	DM
Point estimate, BN	19.5%	7.3%	6.4%	1.4%	2.6%	23.0%	15.0%	7.8%	4.3%	1.4%
Point estimate, DE	16.5%	7.1%	7.1%	1.7%	2.1%	23.8%	15.9%	7.9%	4.8%	1.8%
Monte Carlo SE, BN	0.20%	0.16%	0.16%	0.16%	0.16%	0.20%	0.18%	0.17%	0.16%	0.16%

## Conclusions

Our contributions in this manuscript are to first define sequential and average attributable fractions in a causal framework and second to describe a possible methodology to estimate these quantities based on simulation from causal Bayesian networks. However, it is imperative to describe several caveats to our work. First, assuming the sequential attributable fractions in the Methods section are well defined causal estimands, consistent estimation is only possible under strong assumptions that the assumed DAG is a causal Bayesian network with no missing confounders, and that our modeling assumptions assumed when estimating the component probability disbributions are correct. It is often said that causal inference usually involves unverifiable assumptions [15], and that is certainly the case here. While the causal structure we’ve assumed might correspond to an approximately correct but overly-simplistic model for prospective risk factor development, estimating these relationships in a case-control data-set is problematic due to possible reverse causation. In addition, the use of categorized risk factors (rather than the underlying continuous exposures) may result in inadequate adjustment for confounding. However, these problems are not unique to an approach based on Bayesian networks and will create biases even under simpler (and incorrect) models which only consider direct causal effects that a risk factor has on disease. The second caveat relates to our definitions of attributable fractions through potential outcomes and whether this is in some cases ill-defined. For instance, our definition of the attributable fraction for diet (in Results) involves a hypothetical population where individuals had measured AHEI-diet-scores in the top (or most healthy!) tertile. There are many ways of engineering such a diet, all of which might have differing potential outcomes for stroke and generate diet score values in the desired range (the top tertile of the empirical distribution of diet score among INTERSTROKE participants). Attributable fractions refer to interventions (where a risk factor is removed in a hypothetical population); one could rephrase the problem of the ill-defined potential outcome by saying that the intervention that ‘removes’ the risk factor is not particularly well defined. A school of thought might say that well defined causal effects require a contrast of potential outcomes under well defined interventions [16].

It might at first be thought that the simulation approach using Bayesian networks is in some sense ‘overkill’. After all, one can estimate causal effects and attributable fractions for a single risk factor using a single regression model by using the *g*-formula as described in [6]. Calculating sequential attributable fractions, on the other hand, requires estimating average causal effects for joint interventions and based on the position of these risk factors within the causal graph, more complicated estimation approaches are necessary. For instance, if we want to find the average joint causal effect of an intervention on diet and cardiac factors on stroke (based on the causal DAG given in Fig. 1), one might at first try using ‘standardization’, but on closer examination, blood pressure is both a mediator of the effect of diet and a confounder for the effect of cardiac factors, and an adjustment set that includes both excludes or includes diet is prone to bias. This issue can be dealt with using methods like the time-varying g-formula and marginal structural models, as again described in [6], but require extra thought and expertize in their application. Interestingly, our approach motivated by recursive application of the do-operator as described first by Pearl, corresponds closely to a simulation based version of the g-formula in causal structures involving time varying confounding. However, for complex causal structures where an arbitrary set of risk factors are intervened on, the simulation based approach described in this manuscript certainly seems the easiest way to proceed.

## Supplementary information

**Additional file 1** Odds ratios from models in final causal networkR1.

**Additional file 2** Rcode_June19.

## Data Availability

R-code for estimating sequential and average attributable fractions assuming a Bayesian network structure is available as Supplementary material.

## Abbreviations

PAF: Population Attributable Fraction DAG: Directed Acyclic Graph
